# A Retrospective Cohort Analysis of Short-Term Outcomes of the Mycophenolate Mofetil-Based Triple Regimen for the Treatment of Calcineurin Inhibitor-Resistant Childhood Nephrotic Syndrome: A Single-Center Experience

**DOI:** 10.7759/cureus.102690

**Published:** 2026-01-31

**Authors:** Naeem Mumtaz, Aasia Zubair, Madiha Aziz, Sabeeta Khatri, Irshad Bajeer, Ali Asghar A Lanewala

**Affiliations:** 1 Pediatric Nephrology, Sindh Institute of Urology and Transplantation, Karachi, PAK; 2 Pediatric Nephrology, Sindh Institute of Urology and Transplantation, karachi, PAK

**Keywords:** childhood nephrotic syndrome, cni-resistant, mycophenolate mofetil, srns, triple regimen

## Abstract

Background

Idiopathic nephrotic syndrome (INS) is a heterogeneous group of disorders. A variable number of children do not respond to the conventional first line of treatment and are at risk of progression and complications of nephrotic syndrome. There is limited literature on the use of a combination of alternative immunosuppressive agents in children with calcineurin inhibitor (CNI)-resistant nephrotic syndrome (NS). This study is designed to review short-term outcomes of children who have failed to respond to CNI with documented adequate levels and preserved renal function.

Methods

Of the 490 registered children receiving CNI, retrospective data were reviewed for 37 patients who received mycophenolate mofetil (MMF) along with conventional CNI and steroids after demonstration of CNI resistance with adequate drug levels. Using the predesigned proforma, clinical, histopathological, and laboratory parameters were collected. Outcomes were classified as complete, partial, and no response.

Results

The median age of the study participants was 10 years (interquartile range (IQR): 7.75-13.2), with a male predominance of 62%. A total of 24 out of 37 (65%) children responded to the addition of MMF, with half of them achieving complete remission (CR). With the addition of MMF to the conventional regimen, the improvement in serum albumin was statistically significant (OR: 9.29, 95% CI: 1.66-51.74; p = 0.011).

Conclusion

This retrospective study demonstrates the short-term efficacy of MMF-based triple regimen in achieving remission in pediatric CNI-resistant nephrotic syndrome and is a cost-effective treatment option. Larger, prospective studies with longer follow-up are, however, required to confirm these findings.

## Introduction

Idiopathic nephrotic syndrome (INS) is a heterogeneous group of disorders. Approximately 80%-85% respond to treatment with steroids, steroid-sensitive nephrotic syndrome (SSNS), while the rest are resistant to steroid therapy, steroid-resistant nephrotic syndrome (SRNS) [1]. There is consensus among guidelines from different societies that calcineurin inhibitors (CNIs) are the recommended first-line treatment for SRNS, and either complete or partial remission is achieved within the first six months in 60%-80% [2-4]. However, there is a variable number of children who do not respond to the first line of treatment and are at risk of progression and complications of nephrotic syndrome [5].

There are some anecdotal reports on the use of mycophenolate mofetil (MMF) in children who have frequent relapses of nephrotic syndrome while on CNIs or those who do not respond to CNIs. The International Pediatric Nephrology Association (IPNA) and Kidney Disease Improving Global Outcomes (KDIGO) recommend using MMF in those children. The children who do not respond to CNIs can then be treated with rituximab (RTX), cyclophosphamide (CYP), or enrolled in trials of newer experimental drugs [2-4].

There is limited literature on the use of a combination of alternative immunosuppressive agents in children with CNI-resistant nephrotic syndrome (NS). This study is designed to review short-term outcomes of children who have failed to respond to CNI with documented adequate levels and preserved renal function. The primary objective was to assess the efficacy of the addition of MMF and steroids in children who show CNI-resistant NS. The secondary objective was to assess adverse events associated with this triple regimen and identify laboratory parameters that could predict response.

To the best of our knowledge, this study has included the largest number of children with uniform inclusion criteria and treatment regimens. The results of this research will help clinicians choose an alternative treatment regimen for patients with CNI-resistant NS.

## Materials and methods

This retrospective study was conducted at the Department of Pediatric Nephrology of Sindh Institute of Urology and Transplantation (SIUT) after approval from the Institutional Ethical Review Committee (SIUT-ERC-2025/A-527). Using the electronic health record (EHR), data for all patients with INS registered between January 1, 2018, and December 31, 2024, were retrieved and screened for CNI prescription. Of the 490 patients with nephrotic syndrome receiving CNIs, including both steroid-dependent nephrotic syndrome (SDNS) and SRNS patients, we identified 37 patients who had CNI resistance and had received MMF additionally, for a minimum duration of six months, and enrolled them in our study.

Enrolled children were aged 2-18 years with SDNS or SRNS and had received calcineurin inhibitor (CNI), either ciclosporin (CyA) or tacrolimus (TAC), in adequate doses (CyA: 3-5 mg/kg/day and TAC: 0.05-0.15 mg/kg/day). Therapeutic drug levels (100-150 ng/ml for CyA and 5-8 ng/ml for TAC) were maintained for at least six months; however, patients continued to have edema, nephrotic-range proteinuria, and serum albumin levels below 2.5 gm/L, with a preserved eGFR of >60 ml/min/1.73 m^2^. They were then prescribed MMF additionally at a dose of 1000-1200 mg/m²/day in combination with steroids and adjunctive therapy of renin-angiotensin-aldosterone system inhibitors (RAASi). Steroids were given at a dose of 1 mg/kg on alternate days, which was tapered to the lowest effective dose (0.25 mg/kg/EOD) or discontinued.

Children with congenital or infantile NS, secondary NS due to systemic disease, familial NS with affected siblings or first-degree relatives, and renal failure before enrollment in the study were excluded.

A predesigned proforma was used to collect information using the EHR system of the enrolled participants. The proforma included all relevant demographic, clinical, histopathological, and laboratory parameters, including proteinuria on urine dipstick, serum albumin, renal function, prescribed drug regimens, and their side effects, such as GI disturbances, hypertrichosis, deterioration of renal function, and cushingoid appearance. Outcomes were classified as complete response (CR), partial response (PR), and no response (NR).

Standard definitions were used to define SDNS and SRNS [2]. Primary CNI resistance was defined as failure to achieve a complete or partial response to CNI (ciclosporin or tacrolimus) despite adequate levels after six months of therapy [6]. Secondary CNI resistance is defined as an initial response to CNIs followed by relapse on tapering of CNIs and absence of response on reinstitution of the drug with adequate levels [7]. CR was defined as the resolution of edema, absence of proteinuria on dipstick, and serum albumin of >3 gm/L; PR was defined as resolution of edema, +1/+2 proteinuria on dipstick, and serum albumin >2.5 gm/L, and NR was defined as the presence of edema, >+3 proteinuria on dipstick, and serum albumin <2.5 gm/L [8]. The patients were monitored regularly for response using urine dipstick and serum albumin levels. The total leukocyte count was aimed at greater than 4000/mm³. The side effects of drugs were also assessed at each visit. Response was assessed at 3, 6, and 12 months with a minimum follow-up duration of at least six months.

Statistical analysis

All the statistical analyses were done using SPSS, version 27 (IBM Corp., Armonk, NY). Continuous data were analyzed for normality using the Kolmogorov-Smirnov test and represented as mean (standard deviation (SD)) or median with interquartile ranges (IQR). Frequencies were reported as percentages, and continuous and categorical data were analyzed using an independent t-test and chi-square tests, respectively. To identify predictors of response, binary logistic regression analysis was performed, reported as odds ratio (OR) with 95% confidence interval (CI). A p-value of <0.05 was taken as significant.

## Results

Of the 490 registered patients receiving CNIs, 37 (7.5%) patients were identified who did not respond to CNIs despite having adequate drug levels. The median age of the study participants was 10 years (IQR: 7.75-13.2), with a male predominance of 62%. Table 1 summarizes the demographic and clinical details of the study participants.

**Table 1:** Baseline characteristics of study participants Independent t-test and chi-square tests are used for continuous and categorical data, respectively. P-value < 0.05 is considered statistically significant. CR: Complete responder; PR: Partial responder; NR: Non-responder; IQR: Interquartile range; SDNS: Steroid-dependent nephrotic syndrome; SRNS: Steroid-resistant nephrotic syndrome; FSGS: Focal segmental glomerulosclerosis; AKI: Acute kidney injury.

Characteristics	Overall (n = 37)	Responders (CR/PR) (n = 24)	Non-responder (n = 13)	P-value
Age at enrollment, years	3.5 (IQR: 2.2, 5.25)	3.7 (IQR: 2.12-6)	3 (IQR: 2.2-4)	0.19
Age at disease onset, years	10 (IQR: 7.7, 13.2)	10 (IQR: 8-15.3)	11 (IQR: 7-12)	0.82
Gender (male)	23 (62%)	15 (63%)	08 (62%)	0.95
Disease duration, years	06 (IQR: 4.2, 8.5)	5.5 (IQR: 4.1-8.6)	07 (IQR: 4-8)	0.47
Nephrotic syndrome type				
SDNS	09 (24%)	06 (25%)	03 (23%)	0.89
SRNS	28 (76%)	18 (75%)	10 (77%)	
Serum cholesterol (gm/dl) at diagnosis	376 ± 70.4	368.3 ± 66.9	392.2 ± 76.8	0.33
Serum albumin (gm/dl) at diagnosis	1.47 ± 0.45	1.56 ± 0.47	1.31 ± 0.35	0.14
CNI resistance				
Primary	12 (32%)	07 (29%)	05 (38%)	0.71
Secondary	25 (68%)	17 (71%)	08 (62%)	
Serum albumin (gm/dl) at MMF initiation	1.2 ± 0.21	1.47 ± 0.5	1.2 ± 0.21	0.74
Serum albumin (gm/dl) at 3 months	2.53 ± 1.51	2.87 ± 1.13	1.9 ± 0.93	0.012
Serum albumin at (gm/dl) 6 months	2.66 ± 1.21	3.11 ± 1.2	1.84 ± 0.7	0.001
Serum albumin (gm/dl) at 12 months	3.15 ± 1.12	3.64 ± 0.78	1.86 ± 0.81	<0.001
Renal biopsy (FSGS)	11 (30%)	06 (25%)	05 (38%)	0.71
Blood pressure (Normal)	33 (89%)	23 (96%)	10 (77%)	0.77
Drug complications				
Cushingoid facies	15 (41%)	09 (64%)	06 (86%)	0.73
AKI	02 (05%)	02 (08%)	00	0.53
Diarrhea	08 (21%)	05 (20%)	03 (23%)	1.00

A total of 24 out of 37 (65%) children responded to the addition of MMF, with half of them achieving CR. Statistical analysis of clinical parameters to evaluate predicting factors showed no statistically significant differences between responders and non-responders in terms of gender, age at diagnosis, blood pressure, morphology of the glomerular lesions, duration of disease, serum albumin and cholesterol levels at diagnosis, and quantity of proteinuria.

**Figure 1:**
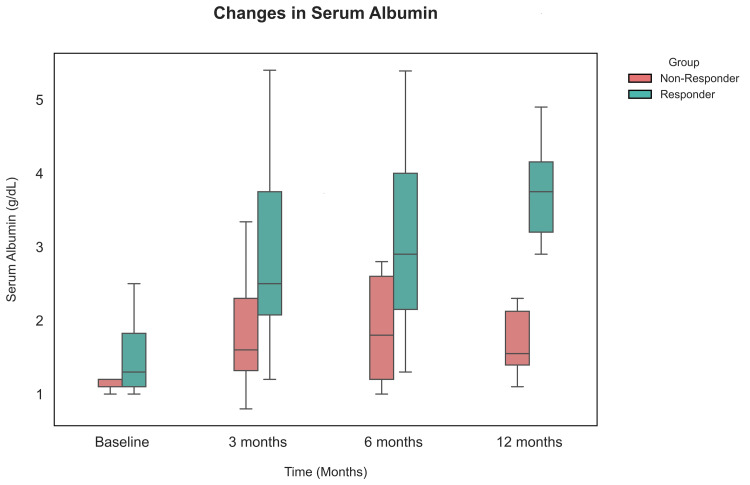
Box plot showing serum albumin trajectory over 12 months from initiation of MMF till 12 months in responders and non-responders MMF: Mycophenolate mofetil.

Serum albumin levels of responders progressively increased after initiation of MMF when measured at 3, 6, and 12 months of therapy, while for the non-responders, although there was improvement after initiation of therapy, it remained less than the desired range. The difference in albumin levels in both groups was statistically significant at all intervals (Figure 1).

Table 2 illustrates logistic regression analysis of gender, disease duration, histopathological diagnosis of focal segmental glomerulosclerosis (FSGS), and serum albumin levels at 3, 6, and 12 months after initiation of therapy as predictors of outcome. With the addition of MMF to the conventional regimen, the improvement in serum albumin was statistically significant (OR: 9.29, 95% CI: 1.66-51.74; p = 0.011), suggesting the efficacy of MMF. Shorter disease duration (<7 years) and FSGS were also associated with a better response; however, this association was not statistically significant.

**Table 2:** Logistic regression test showing predictors of remission and change of serum albumin at regular intervals after MMF addition FSGS: Focal segmental glomerulosclerosis; MMF: Mycophenolate mofetil.

Variables	Odds of Remission (95% CI)	P-value
			Boys	1.04 (0.26, 4.18)	0.95
Disease duration (<7 years)	1.51 (0.36, 6.29)	0.56
FSGS	1.87 (0.44, 7.99)	0.39
Post-MMF serum albumin (3 months)	2.7 (1.13,6.66)	0.02
Post-MMF serum albumin (6 months)	3.70 (1.42, 9.64)	0.007
Post-MMF serum albumin (12 months)	9.29 (1.66, 51.73)	0.01

The most predominant side effect observed with this therapy was gastrointestinal upset, seen in 21% of the patients, half of whom developed acute kidney injury (AKI) due to dehydration. About 5% of the children experienced AKI only, which improved within two weeks of adjusting the dose of CNIs.

## Discussion

With advances in molecular diagnostics, the pathophysiology of nephrotic syndrome is better understood. More than 250 different proteins have been identified in the filtration barrier, and loss-of-function of approximately 50 such proteins has been shown to cause monogenic SRNS [9]. There is a variable response to different medications that can now be attributed to an autoimmune process. The discovery of specific antibodies against filtration barrier proteins, such as nephrin and podocin [10], has opened a new era in understanding the pathogenesis of this unpredictable response. Until specific treatment can be tailored to individual cases, clinicians must treat empirically.

Since the majority of children respond to first-line therapy (steroids for SSNS and CNI for SRNS), the number of children with CNI resistance in other studies is low. Therefore, there is limited evidence on the response to a combination of immunosuppressant medications in a uniform cohort. Nikibakhsh et al. in 2011 reported their retrospective experience of 37 Iranian children with SRNS out of a total of 90, seen between 2002 and 2008. Cyclosporin resistance was based on six months of therapy with a 5 mg/kg/day dose, and patients were given 30 mg/kg/day of MMF. The total response rate was 56% in these children. This study had the largest cohort of INS children, of which 23 demonstrated CNI resistance. However, they did not check the trough levels of cyclosporin and glomerular filtration rates (GFRs) before labeling them CNI resistant after six months of therapy. Their response rate was slightly lower than ours (56% vs 65%) [11].

In another study of 13 CNI-resistant patients with adequate trough levels, Gaur et al. have reported 86% sustained remission over a year by giving rituximab and MMF [12]. Okada et al. gave MMF to children with FRNS who continued to have relapses while on CyA and reported improvement in 11 patients [13]. Another study by Yang et al., a multicenter open-label RCT using MMF in SDNS children on CyA, did not show a significant benefit [14].

Similarly, Wu et al. have reported a total of 18 patients and randomly assigned five patients to MMF and others to cyclophosphamide and leflunomide. They included not only CNI-resistant patients but also those who were CNI sensitive and had frequent relapses. An improvement was noticed in all three groups, and no significant difference was seen between the groups [15]. In our study, we included only those children who received MMF along with therapeutic levels of CNI and concomitant steroids at 1 mg/kg/every other day.

The side effect profile of MMF reported is usually mild, and the most reported side effects are GI-related, across all age groups [16,17]. It was reported in 21% of our children, and one-fourth of these children also had an episode of AKI. The AKI in these patients could be attributed to the hypovolemia induced by diarrhea or concurrent use of CNI. However, the severity of the side effects did not result in treatment withdrawal in any of the children over one year. Our study documented CNI-related AKI and cushingoid facies secondary to steroid use. Wu et al. documented similar steroid and CNI-related adverse events in their population [15].

MMF is a potent immunosuppressive medication that works as an antimetabolite targeting rapidly proliferating cells. It has shown the best results in antibody-mediated diseases, such as acute rejections post-transplant [18]. Sinha et al. have reported inferior response to MMF in SRNS as monotherapy [19]. The discovery of antibodies against proteins like nephrin, leading to nephrotic syndrome, has helped us to hypothesize the excellent response to a subset of children with nephrotic syndrome to antimetabolites and monoclonal antibodies like rituximab [20].

Serum albumin not only has a role in the diagnosis of INS, but it is also used as a marker of remission in SRNS patients on second-line immunosuppressive (IS) regimens [1,16]. Different studies define remission as complete or partial based on serum albumin and urinary protein quantification [1,19]. Our study has identified statistically significant improvements in serum albumin in responders and non-responders. Although the serum albumin levels of non-responders were below 2.5 gm/dl, they did increase from baseline, suggesting the efficacy of the triple regimen.

The retrospective design of this study did not allow us to screen for antibodies in this cohort, which can be considered a primary weakness of this research; however, it will be used as a direction for future projects. If this subset of children with SRNS who do not respond to CNIs alone and also have antibodies to podocyte proteins, it is possible to stratify children with NS at the onset and tailor their therapy accordingly. The non-availability of mycophenolic acid (MPA) levels, especially in those patients who had a PR to the addition of MMF in their treatment regimen, short-term follow-up, and lack of a comparator group are a few other weaknesses. Uniform inclusion criteria, documented therapeutic CNI levels, and the largest pediatric CNI-resistant cohort in our region are strengths of this study. While most SRNS patients respond to the first-line recommended CNI, ineffective reduction of proteinuria and poor long-term outcomes remain challenges in the subset of patients who demonstrate CNI resistance. Alternative treatment strategies must be assessed to achieve outcomes in the face of treatment resistance. This study evaluates the short-term outcomes of triple drug therapy in pediatric CNI-resistant nephrotic syndrome.

## Conclusions

This retrospective study demonstrates the short-term efficacy of MMF-based triple regimen in achieving remission in pediatric CNI-resistant nephrotic syndrome and is a cost-effective treatment option. Larger, prospective studies with longer follow-up are, however, required to confirm these findings.
